# Acute Post-infectious Glomerulonephritis in Children Admitted to a Tertiary Care Hospital: A Descriptive Cross-sectional Study

**DOI:** 10.31729/jnma.8554

**Published:** 2024-04-30

**Authors:** Mandira Shrestha, Romila Chimoriya, Amrit Dhungel, Sujit Koirala, Bijay Basnet, Rohit Bhatta

**Affiliations:** 1 Department of Paediatrics, Nepal Medical College and Teaching Hospital, Jorpati, Kathmandu, Nepal

**Keywords:** *children*, *edema*, *hypertension*, *postinfectious glomerulonephritis*, *poststreptococcus glomerulonephritis*

## Abstract

**Introduction:**

Post infectious glomerulonephritis remains the most common cause leading to the majority of hospital admissions in children of developing countries like ours. The aim of our study was to find the prevalence of post infectious glomerulonephritis, study the clinical profile, biochemical changes and its complication in children admitted in a tertiary care hospital of Nepal.

**Methods:**

This descriptive cross-sectional study of children admitted at a tertiary care hospital was done from May 2020 till May 2023. A census sampling method was used and sample of 1554 children was taken. Detailed socio demographic data, clinical findings and laboratory investigations were done. Data analysis was done using SPSS software and the results obtained are shown in the form of frequencies along with percentages.

**Results:**

Among 1554 patients, the prevalence of acute post-infectious glomerulonephritis was found to be 63 (4.05%) (3.07-5.03 at 95% Confidence Interval). The mean age of the patients was 9.06±3.48 years. Antistreptolysin O titer was raised in 34 (54%) patients, while low serum C3 was observed in 39 (61.90%) patients with acute post-infectious glomerulonephritis.

**Conclusions:**

Acute post-infectious glomerulonephritis (APIGN) remains a notable health concern in children, particularly in developing countries like Nepal. This highlights the need for ongoing surveillance, prevention strategies, and effective management protocols to address this burden effectively.

## INTRODUCTION

Acute glomerulonephritis syndrome (AGN) is mainly due to primary renal cause or secondary illness as infectious agents.^1,2^ Post Infectious Glomerulonephritis (PIGN) is one of the major causes of the AGN, among its, Poststreptococcal glomerulonephritis (PSGN) is the most common infectious agent, and mainly caused by Group A phemolytic streptococci.^3,4^

Around 470,000 cases of PIGN occur annually with 97% occurring in developing countries. Nowadays the incidence of PSGN has been decreased in the industrialized countries, while in developing countries like ours is still a crucial public health issue.^5^ Some studies from Nepal on AGN in children reported that AGN 3.1% of annual paediatric admissions.^6-9^

Patients with AGN may be asymptomatic and clinical features differ from microscopic hematuria to typical pictures of acute nephritic syndrome. The aim of our study was to find the prevalence and clinical profile of PIGN, biochemical changes and its complication in children admitted in Nepal Medical College and Teaching Hospital (NMCTH).

## METHODS

This hospital based retrospective descriptive crosssectional study of children admitted at NMCTH was conducted from May 2020 till May 2023. Ethical approval for the study was taken from the Institutional review committee of NMCTH (Reference number: 06080/081). Data was collected from May 2023-Nov 2023 from medical records. A total of records seen for three years were enrolled for the study. All children less than 14 years of age diagnosed with acute nephritic syndrome who were admitted in the department of paediatrics of NMCTH were included in the study. All children more than 14 years and children with evidence of preexisting renal disease such as Hemolytic Uremic Syndrome (HUS), Henoch-Schonlein Purpura (HSP) and lupus nephritis (LN) were excluded. Detailed socio demographic data, clinical findings and laboratory investigations were done. A census sampling method was used. AGN was characterized by hematuria, edema, hypertension, proteinuria, azotemia and oliguria. Diagnostic criteria of Acute Post-Streptococcal Glomerulonephritis (APSGN) were given as follows.^3^

1. Hematuria and/or proteinuria.
2. Evidence of antecedent streptococcal infection by the presence of any one of:
  - ASO titer > 200 unit/ml
  - Group A beta hemolytic streptococci-positive culture
  - Positive reactions to latex agglutination of group A beta-hemolytic streptococci antigen.
3. Transient low serum complement- C3.
4. No clinical or histological evidence of any other renal disease

Hypertension was diagnosed if blood pressure (BP) values were higher than the 95th percentage for age, gender and height.^10^ Oligouria, hematuria, nephrotic range proteinuria, hypoalbunemia, azotemia was defined as per standard protocol. Glomerular filtration rate (GFR) was calculated according to the Schwartz.^11^ Radiological investigations included ultrasonography of kidney, ureter, and bladder and chest x-ray done to watch for complications and renal biopsy (only if indicated) was done. A socio-demographic (name, age, sex, weight at admission, address, socio economics condition), clinical symptoms and signs, diseases that preceded glomerulonephritis, clinical course of the disease, laboratory tests performed during hospital stay, complications and treatments were collected.

Data will be collected and analyzed using SPSS (Statistical Package for the Social Science). Statistical analysis was performed by descriptive method, by the use of frequency, percentage, mean, median, standard deviation and range as per the nature of data. Data was analyzed by using Microsoft Excel and SPSS.

## RESULTS

A total of 1554 patients were admitted to the Paediatric ward. Among 1554 patients, the prevalence of acute post-infectious glomerulonephritis was found to be 63 (4.05%) (3.07-5.03 at 95% Confidence Interval). Among these patients, males accounted for 972 (62.50%), while females comprised 582 (37.45%) of the total admissions. Within the Nephritic syndrome cases, males constituted 41 (65%) and females 22 (35%) (Table 1).

**Table 1:** Demographic profile (n= 1554).

Variables	Number of total patient (n = 1554)	Number of patients with PIGN (n = 63)
**Sex**		
Male	972 (62.50)	41(65)
Female	582 (37.45)	22 (35)
Age group		
≤12 months	801 (51.54)	-
13 month to ≤5 year	447 (28.76)	12 (19)
6 year to ≤10 year	162 (10.42)	25 (39.70)
11 year to ≤14 year	144 (9.27)	26 (41.30)

History of sore throat was present in 19 (30.15%) whereas pyoderma was associated with 9 (14.28%) of cases of PIGN. Family history was found in 1 (1.58%) of cases. The edema of face /and limbs was seen in 45 (71.42%) and hypertension in 41 (65%) (Table 2).

**Table 2:** Clinical features of patients with PIGN (n= 63).^*^

Symptoms/Signs	n (%)
Swelling of face and/or leg Hypertension Red or cola colored urine Pain abdomen Cough Fever Oliguria History of sore throat History of skin lesion Headache Rashes Dyspnea Seizure Joint Pain	45 (71.42) 41 (65) 36 (57.14) 28 (44.44) 26 (41.26) 25(39.68) 12 (19.04) 19 (30.15) 9 (14.28) 4 (6.34) 4 (6.34) 9 (14.28) 5 (7.93) 1 (1.58)

* Multiple Response Question

*

Among 63 cases, ASO titer was raised in 34 (54%) patients, while low serum C3 wasobserved in 39 (61.90%) patient. Deranged renal function was found in 2 (3.17%). In urine analysis, 59 (93.65%) had haematuria, proteinuria in 18 (28.57%) (sub-nephrotic range proteinuria) and 2 (3.17%) had nephrotic range proteinuria. Serum electrolytes (Na, K) were normal in all cases and ANA titer was performed in 9 (14.28%), while 1 had positive titer. Significant pyuria was found in 5 patients and among them, 1 had a positive result in urine cultures sensitivity (Table 3). Anti-DNAse B was done in only one case which was positive. Among 6 cases, throat swab was positive for Group A streptococcus in 3 (50%).

**Table 3:** Biochemical profile of patients with PIGN (n= 63).

Laboratory parameter	n (%)
Haematuria (RBC> 5/hpf)	55 (93.65)
Elevated ASO titer	34 (54)
Deranged renal function	2 (3.17)
Low Serum C3	39 (61.90)
Proteinuria	
Sub nephrotic range	18 (28.57)
Nephrotic range	2 (3.17)
Pyuria (WBC>5/hpf)	5 (7.93)

Among 63 patients, 12 (19.00%) had complications which includes AKI in 1 (1.58%), hypertensive encephalopathy in 4 (6.34%), cardiac failure in 6 (9.52%), and RPGN with CCF and hypertensive encephalopathy in 1 (1.58%) (Table 4).

**Table 4:** Complications of PIGN (n= 63).

Complications	n (%)
Congestive Heart failure Hypertensive encephalopathy AKI RPGN+ CCF+ Hypertensive encephalopathy Mortality	6 (9.52) 4 (6.34) 1 (1.58) 1 (1.58) -

Renal biopsy was performed in 1 (1.58%) case with nephrotic range proteinuria and positive ANA and showed diffuse proliferative glomerulonephritis. All 63 (100%) children had favorable acute outcomes and were discharged from the hospital.

## DISCUSSION

The study found that among 1554 paediatric admissions to a tertiary care hospital in Nepal, APIGN accounted for a prevalence of 4.05%. APIGN predominantly affected males, with swelling of face and limbs, hypertension, and hematuria being common clinical features. Raised antistreptolysin O (ASO) titers and low serum C3 levels supported the diagnosis. Complications, including cardiac failure and hypertensive encephalopathy, were observed in a notable proportion of cases, but no mortality was reported. Despite these complications, all children had favorable acute outcomes and were discharged from the hospital.

Acute post-infectious glomerulonephritis (APIGN) is common in age group of 5-14 year age, predominantly in males with ratio of 1.86:1.3, and is very rare with <5% below 2 years of age while, in the present study the youngest one was 1 year old and the children below 5 years of age is about 19.02%.^6-8,12-14^ These observations were in accordance with the other studies.^7,12-14^ Glomerulonephritis is least common in very early age may be due to immature immune response in early age.^9^ Another explanation for the relatively high rate of toddlers may be the increasing incidence of streptococcal infection or possibly other infectious triggers in this population.^3^

In accordance with previous study,we found higher incidence during winter (54%) followed by summer seasons (30.15%).^3^ In most of the industrialized countries, PIGN incidence has been decreasing significantly during the last few decades, yet it remains a major cause of acute glomerular disease in children around the world.^3^ However, it is still prevalent in our part of world; the major reason for the high incidence of this disease in our country is lower socioeconomic status in the community, where poverty, overcrowding and poor hygienic conditions are prevalent.^6^

In analog to other studies, history of sore throat was the antecedent event in 30.15% of cases and pyoderma in 14.28%. ^3,14^ However, some of the other studies done in Nepal had reported that sore throat infection was less commonly related to PIGN than pyoderma.^6-8^ In the present study, the most common clinical features were swelling of face and limbs (71.42%) followed by hypertension (65%) and gross haematuria (57.14%), which is similar to most of the study.^3,6,8^ While cough and pain abdomen were present in about 40% of cases. Decreased urinary output was less common in our study as compared to another study.^15^ However it was similar to some studies and other clinical features of PIGN as vomiting, dyspnoea, headache, pain abdomen, seizure, and rashes were in accordance to others study.^3,8,12^

In our study 19% of cases encountered, complication of PIGN, the most common were cardiac failure 9.52% followed by hypertensive encephalopathy in 6.34% and RPGN with CCF and hypertensive encephalopathy in 1.58%. Another study had also reported that CCF and Hypertensive encephalopathy were found in 17 % and 3% respectively, while RPGN in 10% which is higher than our study.^8^ The incidence of cardiac complication was also higher in another study that is in correspondence with our study.^6,13^ While the incidence of cerebral complication was higher in comparison to cardiac complications in other studies.^11,15^ Despite the higher incidence of cardiac complications in our study, there was no mortality, similar to other studies. ^6,8,14^ This may be because of early presentation in the hospital, timely management and well control of hypertension.

In our study 54% had positive ASO titer and 61.90% had low serum C3 level which was similar to other studies,while in one of the studies reported 97%,and another one reported 100% of cases had decreased C3 level.^3,8,12-14^ Depressed C3 is a hallmark of PIGN in patients with nephritic syndrome other than non-nephritic causes. Level of C3 varies may be due to the sample being tested on different days, which may vary the day from disease onset of the patients or may be due to genetic diversity in the extent of complement activation.^3^

In contrast to other studies, pyoderma was less common associated with PIGN in our study, still we found ASO positivity to be as high as 77.80% and half of the cases with pyoderma had low serum C3 that was similar to other study. ^6-8,15^ This may be due to that ASO levels differ as per age group and geographical location, so need further evaluation and research. AntiDNAse B titer was done in only one case which found to be highly positive, and throat swab culture were also performed on 6 cases only among them fifty percent had a positive result, however we were not able to send this test to other cases due to financial constraints and unavailability of this test in our centre.

Microscopic haematuria was found in 93.65%, which was similar to other studies. ^3,6-8,12,13^ As per another study, incidence of AKI in patients with PIGN reported 21.40%, while in our study 3.17% of all cases had deranged renal function which was much lower than other previous studies.^15^ The proportion of proteinuria was 31.74% in patient with PIGN, among them 28.57% of all our cases had sub nephrotic range proteinuria and 3.17% of all cases had nephrotic range proteinuria, which was similar to study done by Gunasekaran et al,^13^ while the various studies reported 17%, 14% and 6.67% of all cases had nephrotic range proteinuria, which were much higher than our study.^6,8,16^ This may be because we had excluded all the cases with HSP nephritis and SLE. Among those patients with nephrotic range proteinuria 1 had negative ANA while the other one had positive result, however the case with positive ANA had negative dsDNA and renal biopsy was performed in that case which showed diffuse proliferative glomerulonephritis.

One limitation of this study is its retrospective design, which may introduce biases and limit the ability to establish causal relationships. Additionally, the study's single-centre nature and relatively small sample size may affect the generalizability of the findings to broader populations. The reliance on medical records for data collection may lead to incomplete or inconsistent information, potentially impacting the accuracy of results. Finally, the study's focus on a specific hospital setting in Nepal may not fully capture the diversity of APIGN presentations and outcomes across different regions or healthcare settings, warranting caution in extrapolating the findings to other contexts.

## CONCLUSIONS

This study highlights the ongoing public health significance of this condition, particularly in developing countries. It emphasizes the importance of recognizing APIGN's clinical features, such as swelling of face and limbs, hypertension, and hematuria, supported by diagnostic markers like raised ASO titers and low serum C3 levels. Despite the potential for serious complications like cardiac failure and hypertensive encephalopathy, early detection and management yield favorable outcomes, with no reported mortality in the studied group. Prevention strategies targeting timely treatment of antecedent streptococcal infections and improved hygiene practices are crucial in reducing APIGN prevalence.
